# Effect of the different assays of HbA1c on diabetic patients monitoring

**DOI:** 10.1186/s40200-015-0193-7

**Published:** 2015-08-04

**Authors:** Farideh Razi, Ensieh Nasli Esfahani, Marjan Rahnamaye Farzami, Ali Tootee, Mostafa Qorbani, Soltan Ahmad Ebrahimi, Mehrzad Nahid, Parvin Pasalar

**Affiliations:** Diabetes Research Center, Endocrinology and Metabolism Clinical Sciences Institute, Tehran University of Medical Sciences, Tehran, Iran; Reference Health Laboratory of Iran, Ministry of Health, Tehran, Iran; Department of Community Medicine, Alborz University of Medical Sciences, Karaj, Iran; Non communicable Diseases Research Center, Endocrinology and Metabolism Population, Sciences Institute, Tehran University of Medical Sciences, Tehran, Iran; Massoud Clinical Laboratory, Tehran, Iran; Endocrinology and Metabolism Research Center, Endocrinology and Metabolism Clinical Sciences Institute, Tehran University of Medical Sciences, Tehran, Iran

**Keywords:** HbA1c, Glycemic control, Decision level, Diabetes

## Abstract

**Background:**

HbA1c test is widely used for glycemic monitoring of diabetic patients. This study aimed to evaluate clinical performance of different assays for classification of patients into controlled and uncontrolled group base on ADA recommendations.

**Method:**

A total of 154 samples from patients with diabetes type 2 with HbA1c concentration covering the whole clinical range were analyzed by four commercially methods; D-10 Hb A1c (Bio-Rad Laboratories), Cobas Integra 400 (Roche Diagnostics), NycoCard Reader II (Axis-Shield) and DS5 (Drew Scientific). For each individual assay, patient’s results were classified into controlled and uncontrolled groups (less or more than three decision levels; 6.5 %, 7 % and 8 %) compared to D10 results as reference method. The frequency of each group and also sensitivity, specificity, negative and positive predictive value were estimated.

**Results:**

We found a significant correlation between assays (r: 0.937–0.945). Sensitivity, specificity, and positive and negative predictive values of the evaluated method to identify uncontrolled patients were as follows: 49.2–95.7 %, 86.5–100 %, 89.1–100 %, and 52.9–93.3 %; respectively.

**Conclusions:**

Results show that some HbA1c assays capability to classify diabetic patients according to HbA1c level is still unacceptable.

## Introduction

Measurement of HbA1c concentration can be considered as a useful indicator of glycemic control in diabetic patients. Moreover, it is documented that high HbA1c levels are correlated with development of different diabetes complications. Measurement of HbA1c has more advantages over both assessment of the fasting plasma glucose and glucose tolerance test. It is more convenient, does not require fasting, has increased preanalytical stability, is less likely to differ in different days due to stress or illness [1, 2].

HbA1c testing is also useful in diseases other than diabetes. Van’t Riet in a 10 year follow-up study (Hoorn Study) found that, in non-diabetic persons between 50 and 75 years of age, HbA1c measurement was a superior predictor of 10 year cardiovascular events (fatal and non-fatal) and all-cause mortality, in comparison with fasting and 2 h postprandial glucose [3]. Moreover, the findings of the North American Atherosclerosis Risk in Communities (ARIC) study demonstrated that HbA1c was a better predictor of diabetes and cardiovascular disease in comparison with fasting glucose in patients without diagnosed diabetes [4]. Reducing Hb A1c concentrations to 7 % or below is demonstrated to be correlated with significant decrease in the rate of different microvascular complications of diabetes. Although it is recommended that Hb A1c levels in non-pregnant adults should be kept less than 7 %, more stringent A1c (6.5 %) and mild A1c goals (8 %) may also be recommended for some patient considering their specific situation [2].

Results of one single assessment of HbA1c can provide clinicians with the best determinant of glycemic control in patients and clinical decision-making can be effectively guided by it [5]. Therefore, it can be concluded that the assays for determination of HbA1c levels need to be accurate, precise and consistent to be able to effectively employed by clinicians.

Currently, there exist an exceeding number of 30 different HbA1c assays on the market and they all perform based on two different principles, one of them being the separation of Hb fractions, and the other, on chemical reaction. It is noteworthy however that all of the mentioned assays need to be standardized based on National Glycohemoglobin Standardization Program (NGSP) [6–8].

When choosing an HbA1c measurement method, laboratories should first analyze the method in terms of its being NGSP certified (potential for optimal performance) and then assess proficiency testing data (both current and past surveys) to predict the actual performance of it. Although selection of a NGSP certified method which has successfully passed proficiency testing is very important, it does not still does not guarantee optimal performance in all laboratories [9].

Despite considerable achievements of NGSP in terms of validity of different HbA1c techniques, it is documented that the existing variability among NGSP-certified methods can still undermine clinical diagnostic value of HbA1c testing [10, 11].

The current study is designed to assess discrepancies between HbA1c results obtained by D10-HPLC as reference method and other evaluated assays to determine the diversity in categorization of individual patients in regard to their glycemic control (HbA1c less or more than three decision level; 6.5 %, 7 % and 8 %). We assessed the variability of the results obtained from different techniques in two groups: those with controlled blood glucose levels, and the patients with uncontrolled glucose concentrations.

## Materials and methods

A total of 154 samples from diabetic patients with HbA1c concentration covering the whole clinical range, were obtained through venipuncture into sterile tubes containing the EDTA K_2_ and tested with 4 assays within 2 days. No more inclusion criteria were used.

It needs to be mentioned that the study was approved by the Ethics Committee of Endocrinology metabolism Research Institute and all participants gave written informed consent.

HbA1c measurement was performed concurrently in all cases by four commercially available assays:

1. D-10 Hb A1c (Bio-Rad Laboratories, Hercules, CA), Ion exchange HPLC method
2. Cobas Integra 400 (Roche Diagnostics, Mannheim, Germany), Immunoassay
3. NycoCard Reader II, (Axis-Shield, Oslo, Norway), Boronate affinity chromatography
4. DS5, (Drew Scientific, Le Rheu, France), Ion exchange chromatography

Of these, three methods are currently certified by the National Glycohemoglobin Standardization Program (D-10 Hb A1c, Cobas Integra 400 and NycoCard Reader II).

According to recommendations from the American Diabetes Association (ADA), three decision level for diabetic patients monitoring (6.5 %, 7 % and 8 %) were considered and patients were classified based on their glycemic status by each method (HbA1c < cut off and HbA1c ≥ cut off). After calculation of frequency of each group for each method, sensitivity, specificity, and positive and negative predictive values of the different method were estimated. D10 was considered as the reference method. Total precision of each assay was also evaluated using CLSI EP15-A2 protocol [12].

Data were processed with the statistical software, SPSS version 15. Mean and Standard Deviation, p value and correlation coefficient were calculated. Sensitivity, specificity, and positive predictive values (PPV) and negative predictive values (NPV) of the three assays were estimated.

## Results

Total precision (in term of CV%) were less than 1.6 %, 1.0 %, 3.1 % and 3.3 % for D10, cobas integra 400, Nycocard reader II and DS5 respectively. The mean ± SD value of HbA1c was significantly higher when measured by D10 (7.59 % ± 1.43 %) than when estimated with the Nycocard reader II (6.87 % ± 1.17 %, r: 0.937, P < 0.05) and DS5 (6.69 ± 1.47 %, r: 0945, P < 0.05) and almost same compared to cobas integra 400 (7.66 ± 1.45, r: 0.944, P: 0.06). For each individual assay, patient’s results were classified into controlled and uncontrolled groups compared to D10 results and the frequencies of each group were estimated (Tables 1, 2, 3). In three cut points when using Nycocard, 17.1 %, 26.5 % and 50.8 % of patients could be misclassified as controlled. The same results (27.4 %, 33.3 % and 46 %) were found when using DS5. The results were less than 9.5 % for cobas integra. Sensitivity, Specificity, Negative and positive predictive value were shown in Table 4.

**Table 1:** Classification of patients at HbA1c level 6.5 %

	D10		
Cobas Integra 400	<6.5	≥6.5	Total
<6.5	34	5	39
≥6.5	3	112	115
Total	37	114	154
Nycocard reader II	<6.5	≥6.5	Total
<6.5	37	20	58
≥6.5	0	97	97
Total	37	117	154
DS5	<6.5	≥6.5	Total
<6.5	36	32	68
≥6.5	1	85	86
Total	37	117	154

**Table 2:** Classification of patients at HbA1c level 7 %

	D10		
Cobas Integra 400	<7	≥7	Total
<7	45	7	52
≥7	7	95	102
Total	52	102	154
Nycocard reader II	<7	≥7	Total
<7	52	27	81
≥7	0	75	75
Total	52	102	154
DS5	<7	≥7	Total
<7	52	34	86
≥7	0	68	68
Total	52	102	154

**Table 3:** Classification of patients at HbA1c level 8 %

	D10		
Cobas Integra 400	<8	≥8	Total
<8	84	6	90
≥8	7	57	64
Total	91	63	154
Nycocard reader II	<8	≥8	Total
<8	90	32	122
≥8	1	31	32
Total	91	63	154
DS5	<8	≥8	Total
<8	90	29	119
≥8	1	34	35
Total	91	63	154

**Table 4:** Sensitivity and Specificity of three assays at different Cut Point Compared to D10 Result

	Sensitivity %	Specificity %	PPV %	NPV %
A1C level 6.5 %				
Cobas Integra 400	95.7	91.9	97.4	87.2
Nycocard reader II	82.9	100.0	100.0	64.9
DS5	72.6	97.3	98.8	52.9
A1C level 7 %				
Cobas Integra 400	93.1	86.5	93.1	86.5
Nycocard reader II	73.5	100.0	100.0	65.8
DS5	66.7	100.0	100.0	60.5
A1C level 8 %				
Cobas Integra 400	90.5	92.3	89.1	93.3
Nycocard reader II	49.2	98.9	96.9	73.8
DS5	54.0	98.9	97.1	75.6

## Discussion

The finding of our study demonstrated that although different measurement methods for HbA1c concentrations show very good correlation but might be different in patients with different glycemic control goals. This variation in patients, in turn, might cause confusion in clinicians and affect the proper decision-making.

In our study, Nycocard reader II and DS5 were less sensitive than cobas integra at three cut points: 6.5 %, 7 %, and 8 %. When Nycocard or DS5 were used, 17.1 %–50.8 % and 27.4–46 % of patients were mistakenly identified as the control group. Cobas integra 400 had best ability to detect patients who their results were more or less than predefined decision level and only 4.3 %, 6.9 % and 9.5 % could be underestimated. Fortunately positive predictive value for all evaluated assay was more than 89 % which means the results more than decision level are much more reliable.

In similar study designed by García-Alcalá et al., the authors evaluated the performance of an immunoturbidimetric inhibition assay (Dimension, Dade Behring) and compared it with the HPLC method (Biorad D10). Their findings demonstrated sensitivity and specificity of 78 % and 88 %, respectively, in screening patients with HbA1c levels more than 7 % [5]. In the present study, we observed that immunoassay method (cobas integra) demonstrated sensitivity and specificity of 93.1 % and 86.5 % respectively at HbA1c level 7 %. In a study designed by Schwartz et al., point-of-care testing devices (POCT) were investigated and the results were compared with that of the central labs with which all of them were aligned to DCCT *(*Diabetes Control and Complications Trial) and NGSP. Their findings demonstrated that the sensitivity of POCT devices in detecting patients with HbA1c levels higher than 7 % was 81.8 % [13]. In the current study, the sensitivity of Nycocard reader II as a POCT device was estimated about 73.5 %. Few studies have been done about HbA1c assays performance in Iran with the main focus on persistence of bias in different assay. Karami study showed unacceptable bias in four HbA1c assays including Nycocard [14]. The same results have been achieved by Keramati et al. study which evaluated five HbA1c assays including Nycocard and DS5 [15].

Our results show that being NGSP certified is not enough and the more attention to proficiency testing results and also validation of assays in each laboratory is recommended.
